# Implementation of the graduated compression as an adjunct to pharmaco-thromboprophylaxis in surgery trial results across the UK

**DOI:** 10.1177/02683555221090781

**Published:** 2022-04-24

**Authors:** Rebecca Lawton, Joseph Shalhoub, Alun H Davies

**Affiliations:** Section of Vascular Surgery, Department of Surgery and Cancer, 4615 Imperial College London, London, UK

**Keywords:** venous thromboembolism, graduated compression stockings, surgery, graduated compression as an adjunct to pharmaco-thromboprophylaxis in surgery

## Abstract

**Objectives:**

This study aims to examine uptake and dissemination of a National Institute for Health Research (NIHR) Health Technology Assessment (HTA)–funded trial – Graduated compression as an Adjunct to Pharmaco-thromboprophylaxis in Surgery (GAPS) (project number: 14/140/61) amongst health professionals in the UK. This study aims to evaluate the impact of the trial on venous thromboembolism (VTE) prevention policies 7 months after publication.

**Method:**

A 12-question online survey emailed to 2750 individuals via several vascular societies, 34 VTE Exemplar Centre leads and 1 charity over a 3-month period.

**Results:**

In total, 250 responses were received; a 9.1% response rate. Over half of all respondents (52.4%) had read the GAPS trial results prior to completing the survey. Precisely, 77.1% said their hospital had not yet made changes or did not intend to make changes to local hospital VTE policy based on the GAPS trial.

**Conclusions:**

Findings must be interpreted in the context of the low response rate. Further in-depth interviews would aid understanding of barriers to implementing change.

## Background

It is known that patients undergoing elective surgical procedures are at increased risk of venous thromboembolism (VTE) in the absence of proper administration of thromboprophylaxis.^1^ In recent years, the evidence base has contested the use of graduated compression stockings (GCS), and it was in this context that the Graduated Compression as an Adjunct to Pharmacothromboprophylaxis in Surgery (GAPS) trial was conducted. GAPS investigated the adjuvant benefit of GCS in VTE prevention,^2^ randomising 1905 participants between 2016 and 2019. The GAPS findings indicate that GCS may be unnecessary for the majority of patients undergoing elective surgery.^2^ The aim of this simple survey was to evaluate the impact of the GAPS trial results on clinical practice and understand where there may be inconsistencies in uptake.

## Methods

The survey was designed using the Qualtrics platform and consisted of 12 questions (see Appendix). It was distributed to members of the charity Thrombosis UK (*n* = 1541 healthcare professionals), the Association of Surgeons of Great Britain and Ireland (ASGBI; *n* = 900), the European Venous Forum (EVF; *n* = 160), leads of the UK VTE Exemplar Centres (*n* = 34) and the Society of Vascular Nurses (SVN; *n* = 115) via in-house distribution lists. Responses were collected mid-September to mid-December 2020. Where responses were incomplete, the number of respondents per question is given as denominators with corresponding percentages.

## Results

**Figure 1.**
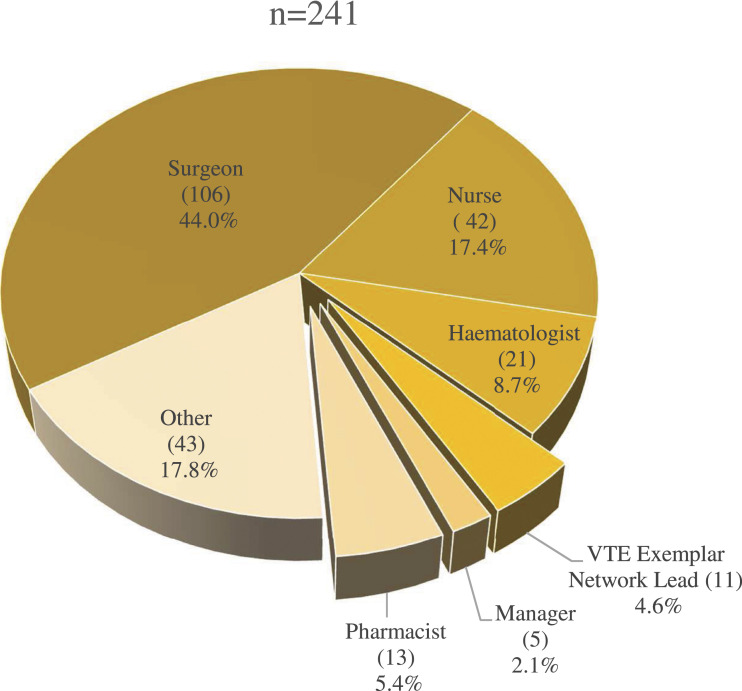
Pie chart showing the roles of survey respondents.

Of the 2750 individuals to whom the survey was distributed, 250 responded, a 9.1% response rate. Most respondents (131 of 250; 52.4%) indicated they had previously read the results of GAPS, with 217 of 250 (86.8%) indicating the results were relevant to their clinical practice. The distribution of respondent profession is shown in Figure 1.

In total, 49 of 214 respondents (22.9%) stated that their hospital had already made changes or planned to make changes to local VTE policy based on the GAPS trial, compared to 165 of 214 (77.1%) who indicated that no changes were planned. For those making changes, we asked what plans had been made or proposed. The majority, 15 of 34 (44.1%), indicated that their hospital planned to stop supplying stockings to surgical patients assessed as being at moderate or high risk of VTE, and 14 of 34 (41.2%) planned to review local VTE policy. Other changes were described by 5 of 34 (14.7%) in free text responses.

We asked if any changes had been implemented already, 15 of 51 (29.4%), indicated that changes would occur in the next 6 months. We asked respondents whether their personal views on the implementation of the GAPS trial results were aligned with those of their employer, 99 of 166 (59.6%) answered ‘yes’ and 67 of 166 (40.3%) answered ‘no’. For those who answered no, we asked them to explain in what ways their views differed. A total of 44 provided an answer, represented by the following themes: Perceptions that the organisation does not want to implement study results or evidence-based medicine, 30 of 44 (68.2%); The views of the organisation are unknown, 3 of 44 (6.8%); The organisation is too slow to implement change, 6 of 44 (13.6%); and Other reason provided, 5 of 44 (11.4%).

## Discussion

This simple survey provides insight into the uptake of the GAPS results amongst health professionals working in VTE prevention. Given the potential cost saving to the NHS, estimated as more than £60 million per annum in England,^3^ and the potential reduction in adverse events associated with GCS, one might assume rapid changes in hospital policy. However, the majority of those surveyed said they did not intend to make changes based on the results of the trial. Research has shown that changing patient care is a complex process and high-level evidence is not always reflected in practice.^4^ Indeed, it took several years and the establishment of VTE Exemplar Centres to realise widespread change in VTE prevention across the UK.^5^ As 46% of respondents were unaware of the results of GAPS, the survey raises questions as to the general lack of impact of trial results. It also highlights the need for investigators to maximise the utility of studies through detailed dissemination plans. Given the relatively early evaluation of the GAPS study and hospitals prioritising review of COVID-19 studies, we recommend further in-depth qualitative interviews with health professionals.

The strengths of this survey include dissemination to a range of stakeholders across the UK and Europe. Limitations include the response rate of 9.1%. Sending reminders would almost certainly have improved this.

## Supplemental Material

Supplemental Material - Implementation of the graduated compression as an adjunct to pharmaco-thromboprophylaxis in surgery trial results across the UK Click here for additional data file. Supplemental Material for Implementation of the graduated compression as an adjunct to pharmaco-thromboprophylaxis in surgery trial results across the UK by Rebecca Lawton, Joseph Shalhoub and Alun H Davies in Phlebology

## References

[1] Michota F . Venous thromboembolism: epidemiology, characteristics, and consequences. Clin Cornerstone 2005; 7(4): 8–15. 16758647 10.1016/s1098-3597(05)80098-5

[2] Shalhoub J Lawton R Hudson J , et al. Graduated compression stockings as adjuvant to pharmaco-thromboprophylaxis in elective surgical patients (GAPS study): randomised controlled trial. BMJ 2020; 369: m1309. 32404430 10.1136/bmj.m1309 PMC7219517

[3] Mandavia R Shalhoub J Head K , et al. The additional benefit of graduated compression stockings to pharmacologic thromboprophylaxis in the prevention of venous thromboembolism in surgical inpatients. J Vasc Surg Venous Lymphatic Disord 2015; 3(4): 447–455.e1. 10.1016/j.jvsv.2014.10.002 26992625

[4] Gupta DM Boland RJ Aron DC . The physician’s experience of changing clinical practice: a struggle to unlearn. Implementation Sci 2017; 12(1): 28. 10.1186/s13012-017-0555-2 PMC5331724 28245849

[5] Gee E . The National VTE exemplar centres network response to implementation of updated NICE guidance: venous thromboembolism in over 16s: reducing the risk of hospital-acquired deep vein thrombosis or pulmonary embolism (NG89). Br J Haematol 2019; 186(5): 792–793. 31168834 10.1111/bjh.16010

