# Supplementary material for: Implementation of the graduated compression as an adjunct to pharmaco-thromboprophylaxis in surgery trial results across the UK
Source: Phlebology. 2022 Apr 24;37(7):540–2. doi: 10.1177/02683555221090781 (PMC9379381; doi:10.1177/02683555221090781)
Supplement: Supplemental Material - Implementation of the graduated compression as an adjunct to pharmaco-thromboprophylaxis in surgery trial results across the UK [file sj-pdf-1-phl-10.1177_02683555221090781.pdf]

Appendix 1 GAPS Implementation survey questions

Q1. Have you read the results of the recently published GAPS trial in the BMJ?https://www.bmj.com/content/369/bmj.m1309

thebmj covid-19 Research Education News & Views Campaigns Jobs

Research

Graduated compression stockings as adjuvant to pharmaco-thromboprophylaxis in elective surgical patients (GAPS study): randomised controlled trial

BMJ 2020 ; 369 doi: https://doi.org/10.1136/bmj.m1309 (Published 13 May 2020)
Cite this as: *BMJ* 2020;369:m1309

- Yes
- No

Q2. The trial concluded that low molecular weight heparin alone is non inferior to a combination of low molecular weight heparin and graduated compression stockings for the prevention of VTE in adult elective surgical patients who were assessed as being at moderate or high risk of VTE. Are the results of the trial relevant to your clinical practice?

- Yes
- No

Imperial College London

Q3. Please select your role:

- ○ Surgeon
- ○ Nurse
- ○ Haematologist
- ○ VTE Exemplar Network Lead
- ○ Manager
- ○ Other

Q4. Has your hospital already made changes or do they plan to make changes to their VTE policy based on the results of the GAPS trial?

- ○ Yes
- ○ No

Q5. If no, why does your organisation not intend to make any changes to their VTE policy?

Q6. what changes have been made or proposed?

- ◯ Stop supplying stockings to elective surgical patients at moderate or high risk of VTE
- ◯ Review local hospital VTE policy
- ◯ Other, please state:

Q7. Have these changes been:

Implemented already. Please indicate for how many months:

Implementation planned. Please indicate proposed time-frame in months

Are your personal views aligned with those of your organisation?

- ◯ Yes
- ◯ No
